# Hospital Festivals and Meetings

**Published:** 1894-02-03

**Authors:** 


### HOSPITAL FESTIVALS AND MEETINGS.

### GERMAN HOSPITAL, DALSTON.

The annual meeting of the governors of the German Hospital, Dalston, was held at the Cannon Street Hotel on January 26th. Baron von Schröder, the chairman, in moving the adoption of the report, called attention to the falling off in the annual income of the hospital, which he attributed to "bad times," and remarked that, had it not been for some unexpected legacies during the past year, there would have been a large deficit in the finances of the hospital. He also alluded to the coming departure for Darmstadt of the Sisters of Mercy who had hitherto worked in the hospital, and whose place, he hoped, would be taken by others from Bielefeld.

The proceedings were here interrupted by the Rev. Dr. Verres, the Roman Catholic pastor to the hospital, who rose to inquire of the chairman whether the hospital was a strictly Protestant institution, or free to all denominations. He put this question because he had been informed that attendance at the Protestant service had, at any rate up to a few months ago, been compulsory on the patients.

This was denied by the Chairman, who said that though the hospital was a Protestant institution, no distinction of creeds was made.

Dr. Verres, proceeding, said that, owing to a regulation which fixed the Protestant service for one day and the Roman Catholic for the next, a priest had been refused permission to visit a dying patient by the secretary, Mr. H. Gülich. This Mr. Gülich flatly contradicted, alleging that access to individual patients was always permitted, and the matter resolved itself into a case of assertion on one side and denial on the other. Some further remarks which Dr. Verres wished to make were overruled by the Chairman.

Baron von Schröder then proposed the election of Mr. Gülich to the combined posts of secretary and superintendent of the hospital, as Mr. Feldmann had been forced, through ill-health, to relinquish the secretaryship, which he had held for thirty-seven years. The motion was carried, but Mr. Loewe wished to know how, with a diminishing income, they were to pay a pension of £200 per annum to Mr. Feldmann, while at the same time they increased, as was proposed, the present secretary's salary from £400 to

£500. He also expressed a hope that the supporters of the hospital would in future be content to regard it as a national and not a denominational institution.

With the latter half of this speech the Chairman entirely agreed, but the former half remained unanswered, Mr. Loewe having given no notice that he intended to move a resolution; and, the usual votes of thanks having been passed, the meeting was declared at an end.

### ITALIAN HOSPITAL.

The annual meeting took place at the Italian Hospital, Queen Square, W.C., on January 27th, with his Excellency Count Tornielli, the Italian Ambassador, in the chair. The report for the past year was read, special stress being laid on the fact that, though designed primarily for Italians, patients of all creeds and nationalities were received; it also reflects credit on the hospital authorities that, in spite of some difficulties in the way of isolation, every effort was made to be ready for the reception of cholera cases at the time when it was feared that there might be many such to deal with. The financial statements not having been audited were reserved for a further meeting.

Sir Joseph Fayrer expressed his opinion that the good work done by the hospital amongst both English and Italians was not widely enough known. He was sure that many Englishmen who knew and loved Italy would be glad to contribute to the funds, were the matter put before them.

The Hon. Secretary, Mr. Serena, then read a letter from the medical officers recommending, until the proposed rebuilding of the hospital could be carried out, various improvements in the out-patient department. Chief among these were the erection of a dark room for the examination of diseases of the eye, ear, and throat; the laying down of a concrete floor in the room allotted to the reception of out-patients, which was described as exceedingly damp, and measures for the better ventilation of the same place. The committee resolved, before taking any measures, to ascertain the cost of the proposed improvements.

### WEST HAM HOSPITAL.

Mr. Passmore Edwards has kindly consented to lay the foundation stone of the proposed new wing of the West Ham Hospital, towards which he has given the munificent sum of £2,000. The ceremony will take place on Thursday, February 22nd next at Half past Three, and it is proposed to have the anniversary hospital dinner on the same day at Seven p.m. at the Guildhall Tavern.

